# Risk factors for concomitant positive midstream urine culture in patients presenting with symptomatic ureterolithiasis

**DOI:** 10.1007/s00240-022-01323-4

**Published:** 2022-04-20

**Authors:** Nico C. Grossmann, Victor M. Schuettfort, Jeannine Betschart, Anton S. Becker, Thomas Hermanns, Etienne X. Keller, Christian D. Fankhauser, Benedikt Kranzbühler

**Affiliations:** 1 Department of Urology, University Hospital Zurich, Zurich, Switzerland; 2 Department of Urology, University Medical Center Hamburg-Eppendorf, Hamburg, Germany; 3 Institute of Diagnostic and Interventional Radiology, University Hospital Zurich, Zurich, Switzerland

**Keywords:** Infected stone, Nomogram, Prediction model, Predictors, Ureter calculus, Urine culture

## Abstract

In patients with symptomatic ureterolithiasis, immediate treatment of concomitant urinary tract infection (UTI) may prevent sepsis. However, urine cultures require at least 24 h to confirm or exclude UTI, and therefore, clinical variables may help to identify patients who require immediate empirical broad-spectrum antibiotics and surgical intervention. Therefore, we divided a consecutive cohort of 705 patients diagnosed with symptomatic ureterolithiasis at a single institution between 2011 and 2017 into a training (80%) and a testing cohort (20%). A machine-learning-based variable selection approach was used for the fitting of a multivariable prognostic logistic regression model. The discriminatory ability of the model was quantified by the area under the curve (AUC) of receiver-operating curves (ROC). After validation and calibration of the model, a nomogram was created, and decision curve analysis (DCA) was used to evaluate the clinical net-benefit. UTI was observed in 40 patients (6%). LASSO regression selected the variables elevated serum CRP, positive nitrite, and positive leukocyte esterase for fitting of the model with the highest discriminatory ability. In the testing cohort, model performance evaluation for prediction of UTI showed an AUC of 82 (95% CI 71.5–95.7%). Model calibration plots showed excellent calibration. DCA showed a clinically meaningful net-benefit between a threshold probability of 0 and 80% for the novel model, which was superior to the net-benefit provided by either one of its singular components. In conclusion, we developed and internally validated a logistic regression model and a corresponding highly accurate nomogram for prediction of concomitant positive midstream urine culture in patients presenting with symptomatic ureterolithiasis.

## Introduction

Urinary sepsis due to upper urinary tract obstruction is most commonly caused by ureteral stones [1] and has a high risk of potentially serious complications such as septic shock and/or disseminated intravascular coagulopathy [2–4]. Thus, the high mortality rate of up to 26% emphasizes the importance for an immediate treatment of concomitant urinary tract infection (UTI) in patients presenting with symptomatic ureterolithiasis. Therefore, accurate identification of these patients could help to guide clinical decision-making and reduce the rate of sepsis development. Moreover, particularly in an era of increasing antibiotic resistances, the number of unnecessary antibiotic treatments could be reduced [5].

However, early diagnosis and treatment of concomitant UTI is challenging, as the incubation time of urine cultures, the gold standard for diagnosis of UTI, usually requires at least 24 h. Therefore, for diagnosis of a concomitant UTI in patients with symptomatic ureterolithiasis, clinicians frequently base their diagnostic and clinical assessment on the subjective interpretation of symptoms, and physical and laboratory findings. However, this approach bears the risk of over- or undertreatment with antibiotics and/or emergent surgical interventions [6]. While previous studies identified several blood and urine laboratory biomarkers for the prediction of UTI, a combined analysis incorporating multiple biomarkers into a multivariable predictive model has not yet been performed [7–18].

We therefore analyzed a consecutive cohort of patients and used a machine-learning-based approach to identify the variables that offer the highest discriminatory power. We hypothesized that this approach will enable the development of a logistic regression model that can accurately identify patients at risk of concomitant UTI by predicting a positive midstream urine culture at time of admission and guide clinical decision-making.

## Patients and methods

### Patient population

We retrospectively reviewed data from a consecutive cohort of patients who presented at our tertiary care emergency department due to a symptomatic ureterolithiasis between 2011 and 2017. Exclusion criteria were missing follow-up, age under 18 years, nephrolithiasis only, reported ongoing antibiotic therapy, anatomical aberrations, solitary kidney, missing laboratory values, chronic bacteriuria due to an indwelling transurethral catheter, ureteric stent, and/or incontinence. The review of the patient cohort included the following variables: patient’s age, gender, symptoms on admission (e.g.; nausea, chill, abdominal pain, flank pain, dysuria, pollakiuria, and gross hematuria), medical conditions (e.g., immunosuppression including organ transplant recipient, diabetes mellitus, human immunodeficiency virus (HIV) infection, and autoimmune/rheumatoid disease), vital signs on admission (e.g., body temperature, blood pressure, and heart rate), laboratory work-up on admission, including extended blood sample analysis [including serum levels of creatinine, C-reactive protein (CRP), hemoglobin, platelets, neutrophils, lymphocytes, and leukocytes], dipstick urine analysis on admission (including urine erythrocytes, leukocyte esterase, pH, and nitrite), as well as radiological findings on admission (e.g., renal pelvis ectasia, perirenal stranding, and fornix rupture). Continuous blood laboratory values were converted to categorical variables (decreased, normal, or increased) based on local laboratory reference ranges. Urine dipstick values were classified as negative/normal or elevated based on the local laboratory report. Urinary tract infection was defined as a positive midstream urine culture with > 10^4^ colony forming units per milliliter (CFUs/mL) excluding the bacteria which are clinically non-relevant or indicating contamination (e.g., *Lactobacillus* spp., *Gardnerella vaginalis*, and/or *Streptococcus* spp.). The study was approved by the local ethics committee (BASEC-Nr. 2017-02036).

### Statistical analysis

Continuous normally distributed variables are expressed as mean ± standard deviation (SD), continuous non-normally distributed variables are presented as median with interquartile range (IQR), and categorical variables are presented as percentage. To simulate external validation and to perform a true model performance evaluation, we randomly divided patients into a training cohort (80%) and a testing cohort (20%). Patients’ characteristics in the training set and testing data set were compared using Wilcoxon rank-sum test, Chi-square test of independence, Kruskal–Wallis test, or Fisher's exact test, as appropriate.

For fitting of the prognostic model, tenfold cross-validation and the least absolute shrinkage and selection operator (LASSO) approach was used to select the most relevant predictors from all available variables. Predictive mean matching was used to impute missing values in the training data set. During the LASSO procedure, a continuously reduced penalty (the sum of the absolute size of the regression coefficients multiplied a tuning parameter (lambda, *λ*) is used to shrink the absolute value of the respective regression coefficients of the assessed variables. Following this approach, some regression coefficients are shrunk to zero. The corresponding variables hold little-to-no discriminatory power and were not used during the fitting of the final model. The optimal value of *λ* was determined by a tenfold cross-validation in the training set. To do so, the area under the curve (AUC) across the cross-validation folds was calculated for different values of *λ*_1.se_. The weight of *λ* that minimizes deviation in the cross-validation is usually determined by *λ*_min_. However, the weight of *λ* that empirically has been shown to create the most parsimonious, but yet informative model, is *λ*_1.se_, which was also used during the fitting of the final model. *λ*_1.se_ is defined as the value of *λ* within one standard deviation of the minimum mean cross-validated error [19]. Variables whose LASSO coefficient were not equal to zero at *λ*_1.se_ were subsequently extracted and used during the fitting the final model. This cross-validation process reduces the risk of overfitting and it is a way of assessing how a model will perform in an independent dataset. In summary, the LASSO procedure allows a machine-learning based variable selection for the fitting of predictive or prognostic models. It has been suggested to be particularly well suited for variables that show high levels of multicollinearity [20, 21].

The selected variables were then used to fit a logistic regression model for prediction of UTI. To evaluate the discrimination ability of this model, the AUC of receiver-operating characteristics (ROC) curves was calculated for both the training and the testing cohort. AUCs were statistically compared using DeLong’s test. The differences between predicted probabilities and the observed proportions were assessed using calibration plots. The Hosmer–Lemeshow test was used to check the goodness-of-fit of the final logistic regression model. Internal validation was performed using 200 bootstrap re-samples as a means of calculating the most unbiased predictive accuracy. Based on the logistic regression models, a nomogram was developed to guide clinical decision-making*.* Finally, the decision curve analysis (DCA) was used to evaluate the clinical net-benefit of the model. All reported p values were two-sided, and statistical significance was set at 0.05. All statistical analyses were performed using R (Version 4.0.3, Vienna, Austria, 2020).

## Results

After applying the exclusion criteria, a cohort of 705 patients was available for analysis. Patient characteristics, clinical and radiological findings, treatment and outcomes of all patients stratified by occurrence of UTI, and training/testing cohort are summarized in Table 1. The laboratory findings and their corresponding reference ranges or cut-off values are displayed in Table 2. In the total cohort, UTI was observed in 40 patients (5.7%). These patients had a significant higher rate of dysuria (28 vs. 12%, *p* = 0.008), higher pulse rate (86 bpm (IQR 64–96) vs. 72 bpm (IQR 64–83), *p* = 0.009), lower diastolic blood pressure [median 82 mmHg (IQR 71–90) vs. 88 mmHg (IQR 78–96), *p* = 0.006], and higher body temperature [36.9 °C (IQR 36.4–37.6 °C) vs. 36.7 °C (IQR 36.4–37.0 °C), *p* = 0.046]. Furthermore, patients with UTI had elevated serum levels of CRP (57 vs 23%, *p* < 0.001), leukocytes (55 vs. 42%, *p* = 0.021), neutrophil granulocytes (63 vs. 39%, *p* = 0.04), and creatinine (40 vs. 22%, *p* = 0.015). On urinary dipstick analysis, UTI patients had significant higher rates of positive nitrite (30 vs. 0.5%, *p* < 0.001) and positive leukocyte esterase (70 vs. 13%, *p* < 0.001). Patients with UTI also required longer inpatient stays compared to patients without UTI (median 4.5 vs 0 days, *p* < 0.001). While the rate of development of sepsis was higher in UTI patients, this did not reach statistical significance (5 vs. 0.8%, *p* = 0.055). Patients with UTI underwent significantly more subsequent surgical interventions (75 vs. 27%, *p* < 0.001) and received more often empirical antibiotic treatment (72 vs. 12%, *p* < 0.001). With the exception of the position of the biggest stone on CT scan as well as hemoglobin and thrombocytes levels, all baseline characteristics and the rate of UTI were equally distributed between the training and the testing cohort.

**Table 1:** Association of urinary tract infection with patient characteristics, clinical/radiological findings, treatment and outcome in 705 patients and stratification by training/testing cohort

Variable	Overall	Urinary tract infection			Test/train cohort		
	*N* = 705	No UTI, *N* = 665	UTI, *N* = 40	*p* value	Training cohort (80%), *N* = 564	Testing cohort (20%), *N* = 141	*p* value
**Characteristics**							
Age	45 (35, 56)	45 (35, 55)	56 (40, 68)	< 0.001	45 (35, 56)	46 (37, 54)	0.7
Sex				0.13			0.7
Female	137 (19%)	125 (19%)	12 (30%)		112 (20%)	25 (18%)	
Male	568 (81%)	540 (81%)	28 (70%)		452 (80%)	116 (82%)	
Pregnancy	139 (20%)	127 (19%)	12 (30%)	0.14	114 (20%)	25 (18%)	0.6
Immuno-suppression	59 (8.4%)	54 (8.1%)	5 (12%)	0.4	48 (8.5%)	11 (7.8%)	> 0.9
Autoimmune/rheumatoid disease	16 (2.3%)	16 (2.4%)	0 (0%)	> 0.9	11 (2.0%)	5 (3.5%)	0.3
Diabetes	41 (5.8%)	36 (5.4%)	5 (12%)	0.075	36 (6.4%)	5 (3.5%)	0.3
**Symptoms**							
Nausea	238 (34%)	226 (34%)	12 (30%)	0.7	190 (34%)	48 (34%)	> 0.9
Vomitus	178 (25%)	171 (26%)	7 (18%)	0.3	134 (24%)	44 (31%)	0.087
Gross hematuria	111 (16%)	105 (16%)	6 (15%)	> 0.9	92 (16%)	19 (13%)	0.5
Dysuria	89 (13%)	78 (12%)	11 (28%)	0.008	71 (13%)	18 (13%)	> 0.9
Pollakisuria	62 (8.8%)	56 (8.4%)	6 (15%)	0.2	48 (8.5%)	14 (9.9%)	0.7
Costovertebral punch sign	393 (56%)	370 (56%)	23 (57%)	> 0.9	312 (55%)	81 (57%)	0.7
Flank back pain	610 (87%)	577 (87%)	33 (82%)	0.6	485 (86%)	125 (89%)	0.5
Abdominal pain	299 (42%)	284 (43%)	15 (38%)	0.6	244 (43%)	55 (39%)	0.4
Inguinal testicular/labial pain	245 (35%)	234 (35%)	11 (28%)	0.4	195 (35%)	50 (35%)	> 0.9
**Vitals**							
Systolic blood pressure	142 (130, 154)	142 (130, 154)	135 (124, 149)	0.082	142 (129, 154)	142 (131, 157)	0.7
Unknown	116	113	3		96	20	
Diastolic blood pressure	88 (77, 96)	88 (78, 96)	82 (71, 90)	0.006	88 (77, 96)	88 (80, 98)	0.3
Unknown	116	113	3		96	20	
Pulse	72 (64, 84)	72 (64, 83)	86 (64, 96)	0.009	73 (64, 83)	72 (64, 86)	0.4
Unknown	114	111	3		95	19	
Temperature	36.7 (36.4, 37.1)	36.7 (36.4, 37.0)	36.9 (36.4, 37.6)	0.046	36.7 (36.4, 37.1)	36.6 (36.4, 37.0)	0.075
Unknown	138	136	2		112	26	
**CT findings**							
Grade of ectasia				0.005			0.2
No ectasia	106 (15%)	103 (16%)	3 (7.7%)		80 (14%)	26 (18%)	
1° ectasia	383 (55%)	366 (55%)	17 (44%)		317 (57%)	66 (47%)	
2° ectasia	177 (25%)	165 (25%)	12 (31%)		136 (24%)	41 (29%)	
3° ectasia	36 (5.1%)	29 (4.4%)	7 (18%)		28 (5.0%)	8 (5.7%)	
Fornix rupture	28 (4.0%)	26 (3.9%)	2 (5.0%)	0.7	20 (3.6%)	8 (5.7%)	0.4
Perirenal stranding	220 (31%)	203 (31%)	17 (42%)	0.2	182 (32%)	38 (27%)	0.3
Position of ureter stone				0.4			0.004
Distal ureter	428 (61%)	407 (61%)	21 (52%)		325 (58%)	103 (73%)	
Middle ureter	94 (13%)	89 (13%)	5 (12%)		82 (15%)	12 (8.5%)	
Proximale ureter	182 (26%)	168 (25%)	14 (35%)		156 (28%)	26 (18%)	
Second ipsilateral stone				< 0.001			0.3
None	392 (56%)	382 (57%)	10 (25%)		310 (55%)	82 (58%)	
Nephrolithiasis	37 (5.2%)	32 (4.8%)	5 (12%)		28 (5.0%)	9 (6.4%)	
Ureterolithiasis	274 (39%)	249 (37%)	25 (62%)		225 (40%)	49 (35%)	
Size of biggest ureter stone (mm)	5.00 (4.00, 6.00)	5.00 (4.00, 6.00)	6.00 (4.00, 8.00)	0.005	5.00 (4.00, 6.00)	5.00 (4.00, 6.00)	0.6
**Patient treatment/outcome**							
Empiric antibiotic treatment	107 (15%)	78 (12%)	29 (72%)	< 0.001	87 (15%)	20 (14%)	0.8
Out or inpatient treatment				< 0.001			0.4
In-patient	289 (41%)	255 (38%)	34 (85%)		226 (40%)	63 (45%)	
Out-patient	416 (59%)	410 (62%)	6 (15%)		338 (60%)	78 (55%)	
Duration inpatient stay days	0.00 (0.00, 3.00)	0.00 (0.00, 2.00)	4.50 (2.75, 7.00)	< 0.001	0.00 (0.00, 3.00)	0.00 (0.00, 3.00)	0.4
Subsequent surgical renal decompression	208 (30%)	178 (27%)	30 (75%)	< 0.001	162 (29%)	46 (33%)	0.5
Development of sepsis	7 (1.0%)	5 (0.8%)	2 (5.0%)	0.055	5 (0.9%)	2 (1.4%)	0.6
Urinary tract infection	40 (5.7%)				30 (5.3%)	10 (7.1%)	0.5

Statistics presented: median (IQR); *n* (%). Statistical tests performed: Wilcoxon rank-sum test; Chi-square test of independence; Fisher's exact test

*UTI* urinary tract infection

**Table 2:** Association of urinary tract infection with laboratory findings in 705 patients and stratification by training/testing cohort

Variable	Overall	Urinary tract infection			Test/train cohort		
	*N* = 705	No UTI, *N* = 665	UTI, *N* = 40	*p* value	Training cohort (80%), *N* = 564	Testing cohort (20%), *N* = 141	*p* value
**Laboratory blood analysis**							
Hemoglobin (range: male: 13.5–17.5 g/dL; female: 12.0–15.5 g/dL)				0.10			0.002
Normal	630 (95%)	595 (95%)	35 (88%)		496 (94%)	134 (99%)	
Decreased	33 (5.0%)	28 (4.5%)	5 (12%)		32 (6.1%)	1 (0.7%)	
Elevated	1 (0.2%)	1 (0.2%)	0 (0%)		0 (0%)	1 (0.7%)	
Unknown	41	41	0		36	5	
Thrombocytes (range 150–450 × 10^9^/ L)				0.2			0.048
Normal	636 (96%)	599 (96%)	37 (92%)		504 (95%)	132 (97%)	
Decreased	21 (3.2%)	18 (2.9%)	3 (7.5%)		20 (3.8%)	1 (0.7%)	
Elevated	7 (1.1%)	7 (1.1%)	0 (0%)		4 (0.8%)	3 (2.2%)	
Unknown	41	41	0		36	5	
Leukocytes (range 4.5–11.0 × 10^9^/L)				0.021			0.5
Normal	378 (57%)	361 (58%)	17 (42%)		300 (57%)	78 (57%)	
Decreased	2 (0.3%)	1 (0.2%)	1 (2.5%)		1 (0.2%)	1 (0.7%)	
Elevated	284 (43%)	262 (42%)	22 (55%)		227 (43%)	57 (42%)	
Unknown	41	41	0		36	5	
Neutrophil granulocytes (range 2.0–7.5 × 10^9^/L)				0.040			0.5
Normal	331 (59%)	320 (60%)	11 (37%)		266 (60%)	65 (55%)	
Decreased	4 (0.7%)	4 (0.7%)	0 (0%)		4 (0.9%)	0 (0%)	
Elevated	230 (41%)	211 (39%)	19 (63%)		177 (40%)	53 (45%)	
Unknown	140	130	10		117	23	
Lymphocytes (range 1.5–4.5 × 10^9^/L)				0.010			> 0.9
Normal	481 (85%)	460 (86%)	21 (70%)		380 (85%)	101 (86%)	
Decreased	54 (9.6%)	46 (8.6%)	8 (27%)		43 (9.6%)	11 (9.3%)	
Elevated	30 (5.3%)	29 (5.4%)	1 (3.3%)		24 (5.4%)	6 (5.1%)	
Unknown	140	130	10		117	23	
CRP levels elevated (> 5 mg/L)	169 (25%)	146 (23%)	23 (57%)	< 0.001	130 (25%)	39 (29%)	0.3
Unknown	42	42	0		35	7	
Creatinine elevated (> 1.2 mg/dl)	153 (23%)	137 (22%)	16 (40%)	0.015	125 (24%)	28 (21%)	0.5
Unknown	43	43	0		38	5	
**Urine dip stick analysis**							
Leukocyte esterase				< 0.001			0.12
Negative	562 (83%)	550 (87%)	12 (30%)		451 (85%)	111 (79%)	
Positive (> 75 Leukocytes/µl)	112 (17%)	84 (13%)	28 (70%)		82 (15%)	30 (21%)	
Unknown	31	31	0		31	0	
Erythrocytes (hemoglobin)				0.065			0.4
Normal	120 (18%)	114 (18%)	6 (15%)		99 (19%)	21 (15%)	
Positive (> 1 mg/L)	554 (79%)	520 (78%)	34 (85%)		434 (77%)	113 (80%)	
Unknown	31	31	0		31	0	
Nitrite				< 0.001			> 0.9
Negative	658 (98%)	630 (99%)	28 (70%)		520 (98%)	138 (98%)	
Positive (> 0.1 mg/dL)	15 (2.2%)	3 (0.5%)	12 (30%)		12 (2.3%)	3 (2.1%)	
Unknown	32	32	0		32	0	
pH value				0.9			0.8
Unknown	31	31	0		31	0	

Statistics presented: *n* (%). Statistical tests performed: Wilcoxon rank-sum test; Chi-square test of independence; Fisher's exact test

*UTI* urinary tract infection, *CRP* C-reactive protein

### Model development, nomogram assessment, and performance evaluation

**Fig. 1:**
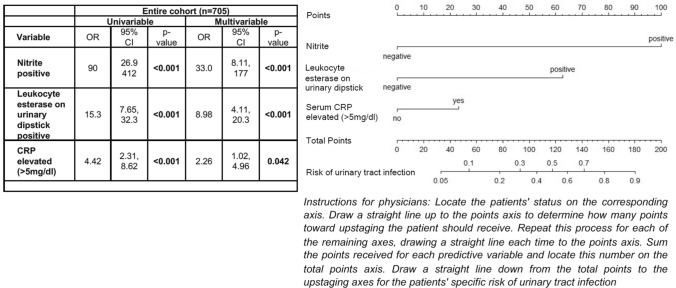
Uni- and multivariable logistic regression model for prediction of positive midstream urine culture (left). The model was fitted using LASSO regression with tenfold cross-validation. Nomogram predicting risk of positive midstream urine culture based on the logistic regression model (*n* = 705, right). *CRP* C-reactive protein, *OR* Odds ratio, *95%CI* 95% confidence interval

**Fig. 2:**
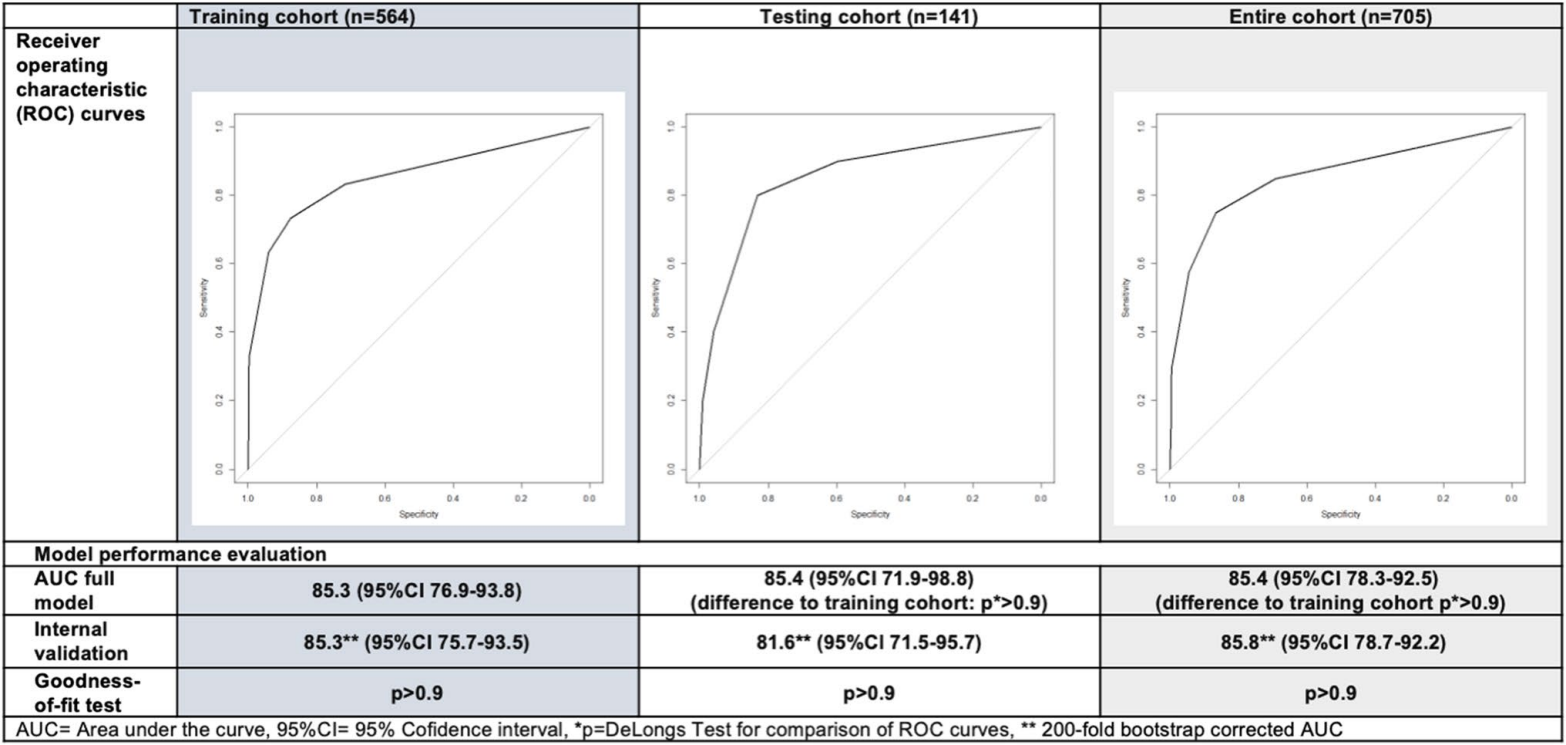
Receiver-operating characteristic curves and model performance evaluation for the prediction of positive midstream urine culture based on the logistic regression model (left: training cohort *n* = 564; middle: testing cohort *n* = 141, right: full cohort *n* = 705). *AUC* area under the curve, *95%CI* 95% confidence interval

From all included variables, LASSO regression selected the variables elevated serum CRP level as well as positive nitrite and positive leukocyte esterase on urinary dipstick analysis in the training cohort for fitting of the model with the highest discriminatory ability. Positive nitrite on urinary dipstick was found to offer the highest discriminatory power for prediction of UTI. The final logistic regression model showed that all three variables remained significantly associated with risk of UTI on multivariable analysis (Fig. 1). Assessment of the nomogram axes indicated that the model demonstrates a wide range of predicted probabilities (5–90%) with positive nitrite contributing the highest number of points. In the training, testing and entire cohort, model performance evaluation showed a 200-fold bootstrap corrected AUC of 85.3% (95% CI 75.7–93.5%), 81.6% (95% CI 71.5–95.7%), and 85.8% (95% CI 78.7–92.2%), respectively (Fig. 2). In the testing cohort, the model demonstrated a negative predictive value of 98.1% and a positive predictive value of 27.6%.

### Model calibration and decision curve analysis

**Fig. 3:**
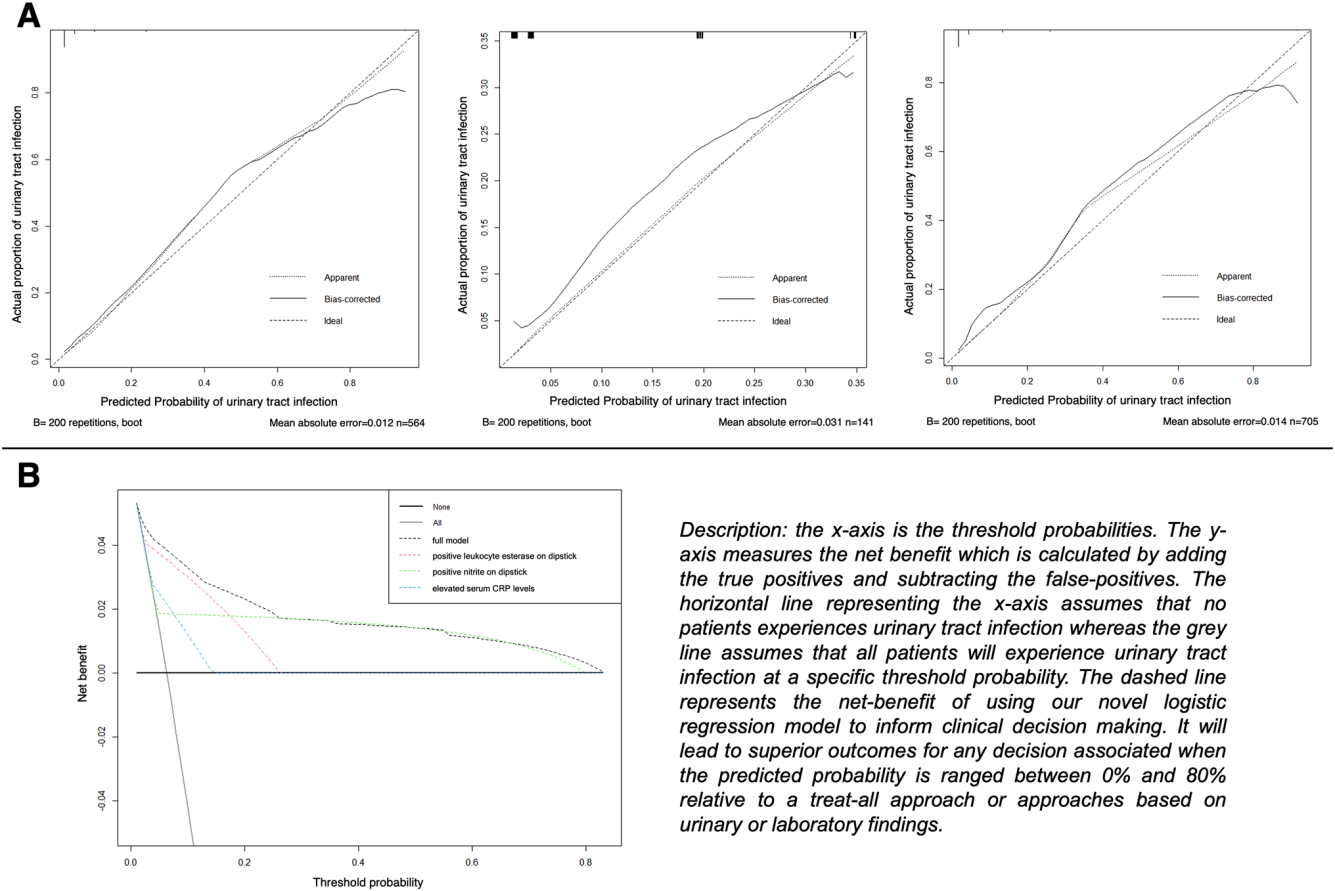
**A** Calibration plots of the logistic regression model predicting of positive midstream urine culture, 200-fold bootstrap corrected (left: training cohort *n* = 564; middle testing cohort, *n* = 141; right entire cohort, *n* = 705). **B** Decision curve analyses for the evaluation of the clinical net-benefit using the novel logistic regression model for prediction of positive midstream urine culture (*n* = 705). *CRP* C-reactive protein

The calibration plot showed that the model showed that the model’s calibration curve ran very close to the diagonal reference line. This suggests near optimal agreement between predicted and observed outcome (Fig. 3A). Correspondingly, the Hosmer–Lemeshow test was insignificant for all cohorts. DCA showed that the model offers a clinical net-benefit relative to the treat-all approach between a threshold of 0–80%. Furthermore, the net-benefit provided by the novel logistic regression model was higher than the net-benefit provided by either one of its singular components (Fig. 3B).

## Discussion

In the current study, we developed and internally validated an accurate and easy-to-use nomogram for prediction of concomitant UTI in patients presenting with symptomatic ureterolithiasis. Using a machine-learning approach, we found that elevated levels of serum CRP as well as positive nitrite and leukocytes esterase on urinary dipstick analysis can accurately identify patients at risk of developing UTI. A 200-fold bootstrap corrected AUC of 81.6% (95% CI 71.5–95.7%) was demonstrated during our internal validation in a cohort that was not used during the development of our model.

As all predictive parameters identified in our model are part of the routine examination in patients presenting with symptomatic ureterolithiasis, we feel that our model is not only easy-to-use but will also be accessible to a broad community of physicians. This is of clinical importance, as there are no existing recommendations on predictive parameters and their thresholds for immediate diagnosis of concomitant UTI. However, individual and subjective interpretation of laboratory markers by clinicians may expose patients to unnecessary empirical antibiotic therapy and/or emergent surgical intervention, while increasing sepsis-related morbidity in case of missed diagnosis.

Results from our multivariable logistic regression analysis confirm the previous univariable findings for early detection of UTI [8, 12, 13, 15]. Nitrite has shown to deliver a low sensitivity of 16.7% but high specificity of 99.5% to diagnose UTI [8, 15]. The low sensitivity of nitrite can be explained by the exclusive detection of Gram-negative rod bacteria [22], which is also reflected in the relatively low probability of approximately 0.35 in our nomogram for an existing UTI when nitrite is found positive. This highlights the need for additional predictors to detect non-nitrite producing bacteria. Indeed, the combination of nitrite and urinary leukocytes esterase as the optimal combination has previously been postulated to rule out UTI with a high reliability [8, 12]. Similarly to urine parameters, elevated CRP levels have been reported to be with an 18-fold increase of UTI in case of obstructive pyelonephritis compared to patients without UTI and dilated renal pelvis [7, 17]. To the best of our knowledge, our model and the corresponding nomogram are the first to incorporate all three parameters to allow accurate prediction of UTI and guide clinical decision-making.

While an experienced urologist will not always require a nomogram to identify patients with concomitant UTI, our findings are still clinically important, as we were able to demonstrate that patients who do not exhibit the findings shown in our nomogram are indeed very unlikely to develop UTI. Hence, with an NPV of 98.1%, our model offers a very reliable method for physicians unfamiliar with obstructive urolithiasis to rule out urinary tract infection. Even though the threshold for early renal decompression should remain low, safe exclusion of UTI could help to reduce the rate of empirical antibiotic therapy, especially in the era of increasing antibiotic resistances. Validated decision-making tools are necessary, as we have found that even in our specialized urological department, 12% of all patients received an antibiotic therapy, which in fact was not necessarily due to negative urine culture. Considering the overall high incidence of symptomatic urolithiasis [23–25], this amounts to a significant amount of unnecessary antibiotic therapy that could be omitted. As genuine clinical applicability of a nomogram has previously been proposed for validated models/nomograms who exhibit AUC/C-indices > 0.75, we feel that our nomogram offers the potential to guide clinical decision-making and could be used by non-urologists for early decision-making and triage [26].

As calibration and validation of nomograms are paramount before the implementation in clinical practice, we performed a statistically rigorous evaluation of the proposed model [27]. Indeed, our model showed nearly perfect calibration properties. Furthermore, the nomogram demonstrates a wide range of predicted probabilities. Finally, the inclusion of only three readily available variables offers a very low level of complexity for our nomogram, suggesting that it is easily reproducible. To allow a realistic model performance evaluation, we aimed to imitate external validation by splitting our cohort into two different cohorts of patients. While true external validation with separate cohorts remains the best assessment of a models accuracy and a crucial step before transferring the models into clinical practice [27], we found that, encouragingly, all results from the training cohort could be reproduced in the testing cohort.

Although our current study uses a statistically rigorous validation and calibration process, several limitations exist. First are the limitations inherent to the retrospective study design. Thus, it is impossible to determine whether laboratory parameters appear to be affected by existing comorbidities and whether a patient has already received an unreported antibiotic treatment prior to evaluation. Second is the single center approach and the limited sample size, as reflected by high odds ratios and wide confidence interval in our multivariable logistic regression model. Third, our endpoint was positive urine culture, which, however, consisted of a single urine collection at the patient admission, giving the potential for missing a positive urine culture during the further clinical course. Additionally, patients with atypical infections or organisms that are hard to culture might have been falsely excluded from our analysis. It should also be considered that a midstream urine culture could be negative, while the urine proximal to an obstructing ureteral stone may be infected. This has been shown in previous studies where urine cultures taken from the renal pelvis were significantly more often positive compared to midstream urine cultures [28]. Furthermore, patients with an infected stone could also have a negative urine culture and might initiate a urinary tract infection during the further clinical course (e.g., by manipulation intraoperatively) [29]. It is therefore important to note that our nomogram predicts a positive midstream urine culture but not a urinary tract infection. Fourth, our results are limited by the failure to control for additional potential predictive parameters such as levels of cytokines or procalcitonin. Finally, radiological findings concerning pyelonephritis or fornix rupture in mostly unenhanced computed tomography are of limited utility. External validation in a larger patient population is needed to verify our findings and help identify patients who require early renal decompression and antibiotic treatment.

## Conclusions

We developed and internally validated a highly accurate, easy-to-use nomogram for prediction of concomitant positive midstream urine culture in patients presenting with symptomatic ureterolithiasis. External validation in a larger patient population is needed to verify our findings and help identify patients who require antibiotic treatment and immediate renal decompression.

## Data Availability

Available upon request to the corresponding author.

## Code availability

Available upon request to the corresponding author.
